# Prevalence of social frailty and its associated factors in the older Chinese population: a national cross-sectional study

**DOI:** 10.1186/s12877-023-04241-1

**Published:** 2023-09-01

**Authors:** Xin Qi, Yingying Li, Jiabin Hu, Lingbing Meng, Ping Zeng, Jing Shi, Na Jia, Xuezhai Zeng, Hui Li, Qiuxia Zhang, Juan Li, Deping Liu

**Affiliations:** 1 Department of Cardiology, Beijing Hospital, National Center of Gerontology, Institute of Geriatric medicine, Chinese Academy of Medical Sciences, No. 1 Dongdan Dahua Road, Dongcheng District, Beijing, 100730 China; 2 Health Service Department of the Guard Bureau of the Joint Staff Department, Beijing, China; 3 The MOH Key Laboratory of Geriatrics, Beijing Hospital, National Center of Gerontology, Institute of Geriatric Medicine, Chinese Academy of Medical Sciences, Beijing, China; 4 Department of Geriatrics, Beijing Hospital, National Center of Gerontology, Institute of Geriatric Medicine, Chinese Academy of Medical Sciences, Beijing, China; 5 China Research Center on Ageing, Beijing, China; 6 Institute of Psychology, Chinese Academy of Sciences, Beijing, China

**Keywords:** Healthy aging, Social frailty, Older adults, Social determinants, Chinese

## Abstract

**Background:**

Social frailty has not been comprehensively studied in China. Our objective is to investigate the prevalence of social frailty among the older population in China, as well as identify relevant factors and urban-rural differences.

**Methods:**

We obtained data from the Fourth Sample Survey of the Aged Population in Urban and Rural China (SSAPUR) database. The study employed a multistage, stratified, cluster-sampling method, recruiting a total of 224,142 adults aged 60 years or older. Participants were interviewed to gather demographic data and information on family, health and medical conditions, health care service status, living environment conditions, social participation, protected rights status, spiritual and cultural life, and health. Social frailty was assessed using the HALFE Social Frailty Index. A score of three or above indicated social frailty.

**Results:**

We analyzed a total of 222,179 cases, and the overall prevalence of social frailty was found to be 15.2%. The highest prevalence was observed among participants aged 75–79 years (18.0%). The prevalence of social frailty was higher in rural older populations compared to urban older populations (19.9% in rural vs. 10.9% in urban, P < 0.0001). In urban areas, women had a higher prevalence than men (11.7% in women vs. 9.9% in men, P < 0.0001), while in rural areas, men had a higher prevalence than women (20.6% in men vs. 19.2% in women, P < 0.0001). Multivariate regression analysis revealed that living in a rural/urban environment (OR 1.789, 95% CI 1.742–1.837), absence of a spouse/spousal presence (OR 4.874, 95% CI 4.743–5.009), self-assessed unhealthy/health status (OR 1.696, 95% CI 1.633–1.761), and housing dissatisfaction/satisfaction (OR 2.303, 95% CI 2.233–2.376) were all significantly associated with social frailty.

**Conclusions:**

Using the HALFE social frailty index, we found a prevalence of 15.2% among older people in China, with the highest prevalence observed in the 75–79 age group. Social frailty was more prevalent in rural areas than in urban areas. Various factors, including spousal presence, housing satisfaction, health status, and urban-rural residential differences, were significantly associated with social frailty. These findings highlight the modifiable and non-modifiable factors that contribute to social frailty among older individuals in China.

**Supplementary Information:**

The online version contains supplementary material available at 10.1186/s12877-023-04241-1.

## Background

In 2015, the World Health Organization (WHO) introduced the concept of healthy aging, defining it as “the process of developing and maintaining the functional ability that enables well-being in older age” [1]. However, as individuals age, they gradually become frail, a condition characterized by reduced physiological reserve capacity and diminished stress resistance due to declines in multiple physiological systems [2]. Frailty serves as a precursor to various adverse health outcomes, exposing individuals to an increased risk of falls, fractures, disability, and morbidity when faced with stressors [3–10]. Due to its impact on healthcare resources and age-related services, frailty has emerged as an urgent public health concern in aging populations. In recent years, frailty has garnered significant attention in the healthcare community due to its potentially detrimental consequences for older individuals and society as a whole. While frailty is often viewed as a physical concept, it is recognized as a multidimensional condition encompassing psychological and social domains as well [4, 11–15].However, studies focusing on the psychological and social aspects of frailty remain relatively limited. Although research on the influence of psychosocial factors on frailty is gaining traction, there are few studies specifically investigating social frailty [11–16]. Therefore, it is crucial to explore the impact of psychosocial factors on frailty, including social frailty.

Social frailty, a distinct type of frailty, has been gaining attention in recent years. It is characterized as being at risk of losing or having already lost essential resources needed to fulfill one or more basic social demands [11, 12, 16–19]. Social frailty not only serves as a risk factor for unhealthy aging but also poses challenges for societies and healthcare systems. Ye et al. showed that demographic characteristics, lifestyle factors and health indicators that might associated with overall frailty as well as three domains of frailty [4]. There are some common risk factors for social and physical frailty, such as female sex, education level, country, physical activity, multi-morbidity, medication risk, and malnutrition, but there are also some differences in risk factors in demographics characteristics, lifestyle and health indicators among the three domains of frailty, and the combination of physical, psychological and social frailty is more likely to contribute to disability and mortality than physical, psychological or social frailty alone [4]. Social frailty and physical frailty are interconnected, with social frailty shown to predict functional impairment, physical frailty, cognitive decline, depression, hospitalization, and mortality among community-dwelling older adults, leading to overall poor health outcomes [5–10, 19].Social frailty is associated with functional disability, physical frailty, and increased dependency on care and assistance from healthcare professionals for older adults [10, 20–24].

Social frailty refers to the absence of social resources, limited social activity, and the inability to fulfill basic social needs. In 2017, Bunt et al. conducted a systematic literature search on social frailty in older adults. Based on the Social Production Functions Theory (SPFT) and factors identified in previous studies, they defined social frailty as a persistent lack of one or more essential resources required to meet basic social needs. Their research also highlighted the importance of considering social behavior, social activities, and self-management skills as components of social frailty [16].

Since its introduction, the concept of social frailty has garnered significant attention. Previous screening tools for social frailty have typically assessed social activities, social support, social networks, loneliness, and living arrangements [5, 7, 10, 18, 25–27]. Consistent with previous studies, we used participants’ living status (whether they lived alone or with others) as an indicator for screening social frailty [8, 9, 28–32]. In China, due to a decrease in the number of children, population aging, and shrinking families, the number of elderly individuals living alone has been increasing. In 2010, there were 18,243,900 older people aged 60 and above living alone in China. From 2000 to 2010, the number of older adults aged 65 and above living alone increased by 6,604,600 (an average annual increase of 660,500), representing an 84.3% increase and an average annual growth rate of 6.3% [33]. Therefore, it is crucial for the country to prepare for the social frailty resulting from population aging and the rising number of older adults living alone. Older adults living alone are susceptible to social isolation, loneliness, and depression due to limited social networks [34]. In our study, we employed the HALFE(“HALFE” is an acronym for the five components: Help, pArticipation, Loneliness, Financial and living alonE)scale as a screening tool for social frailty [35].

The rapid aging of the population in China has made the elderly population one of the largest in the world [36]. Identifying high-risk groups in the early stages of health decline is crucial for maintaining overall health.To develop interventions that promote healthy aging, it is essential to understand the prevalence of social frailty, its related risk factors, and the significance of social aspects in older adults. These aspects play vital roles in improving physical frailty, cognitive decline, disability, overall health, independence, and the need for social support.

A crucial initial step in developing prevention strategies for frailty is to explore the factors associated with it, including identifying groups at risk of becoming frail. However, previous studies have primarily focused on Western populations, investigating various factors influencing social frailty [37–42]. There is a scarcity of research on the factors affecting social frailty specifically in Chinese populations. In our study, we will utilize the data from the Fourth SSAPUR (Sample Survey of the Aged Population in Urban and Rural China) to analyze the factors influencing social frailty, encompassing demographic information, family situations, health status, healthcare and nursing services, economic status, social activities, living environments, and spiritual and cultural aspects.

The main objectives of this study are to investigate the prevalence of social frailty among older Chinese individuals and identify the factors associated with its occurrence. This will enable early intervention in cases of social frailty, facilitating the promotion of healthy aging.

## Methods

### Study population

Data were obtained from the database of the Fourth SSAPUR, conducted by the China National Committee on Ageing in 2015. The survey focused on Chinese citizens aged 60 and above, resulting in the compilation of the largest database of older people in China. The sampling method used in the survey was previously described in a study [35]. The Fourth SSAPUR covered 31 provinces, autonomous regions, municipalities, and the Xinjiang Production and Construction Corps. It encompassed 466 counties (districts), 1864 townships (sub-districts), and 7456 village (residential) committees.The survey questionnaire consisted of nine domains, including demographic information, family situation, health status, healthcare and nursing services, economic status, social activity, living environment, and spiritual and cultural life, which also encompassed psychological status.The questionnaire was designed in both simplified and detailed forms, as outlined in a previous study [35].

The research protocol obtained approval from the National Bureau of Statistics (No. [2014] 87) and the ethics committee of the Beijing Hospital (2021BJYYEC-294-01). Written informed consent was obtained from all participants before completing the questionnaire. The actual number of collected samples was 224,142.

### Procedures

The 4th SSAPUR questionnaire was administered through face-to-face interviews conducted by trained staff. Participants were interviewed to gather demographic data and information about their family, health and medical conditions, healthcare service status, living conditions, social participation, protected rights status, and spiritual and cultural life. In addition, interviewers evaluated the participants’ health (Table S1).The data of the 4th SSAPUR is not publicly available. All procedures were performed in accordance with relevant guidelines.

### Definition of social frailty

The concept of social frailty encompasses five aspects: inability to help others, limited social participation, loneliness, financial difficulty, and living alone. To measure the ability to help others, participants were asked if they had been able to assist their friends or family within the past 12 months. A response of “no” was scored as 1. Limited social participation was assessed by asking participants if they had engaged in any social or leisure activities in the previous 12 months.A response of “no” was scored as 1. Loneliness was measured by a single question: “Do you feel lonely?“ A response of “Yes” was scored as 1. The financial situation was categorized into five grades: very wealthy, relatively wealthy, basically enough, relatively difficult, or very difficult. Financial difficulty was scored as 1 if participants reported a “relatively difficult” or “very difficult” financial situation. Living alone was scored as 1 if participants lived alone. The acronym “HALFE” represents the five components: Help, pArticipation, Loneliness, Financial difficulty, and living alonE. The total score on the HALFE scale ranges from 0 to 5, with a total score of 3 or more indicating social frailty.

### Statistics analysis

Characteristics of subjects with and without social frailty were compared using one-way ANOVA tests and Chi-square tests. Logistic regression was employed in multivariable models to estimate the adjusted odds ratios and 95% confidence intervals (95%CI) of variables associated with social frailty. A p-value < 0.05 was selected as the threshold for statistical significance. All statistical analyses were conducted using SPSS 24.0 (IBM Corp., Armonk, NY, USA).

## Results

### Social frailty versus non-social frailty

**Fig. 1:**
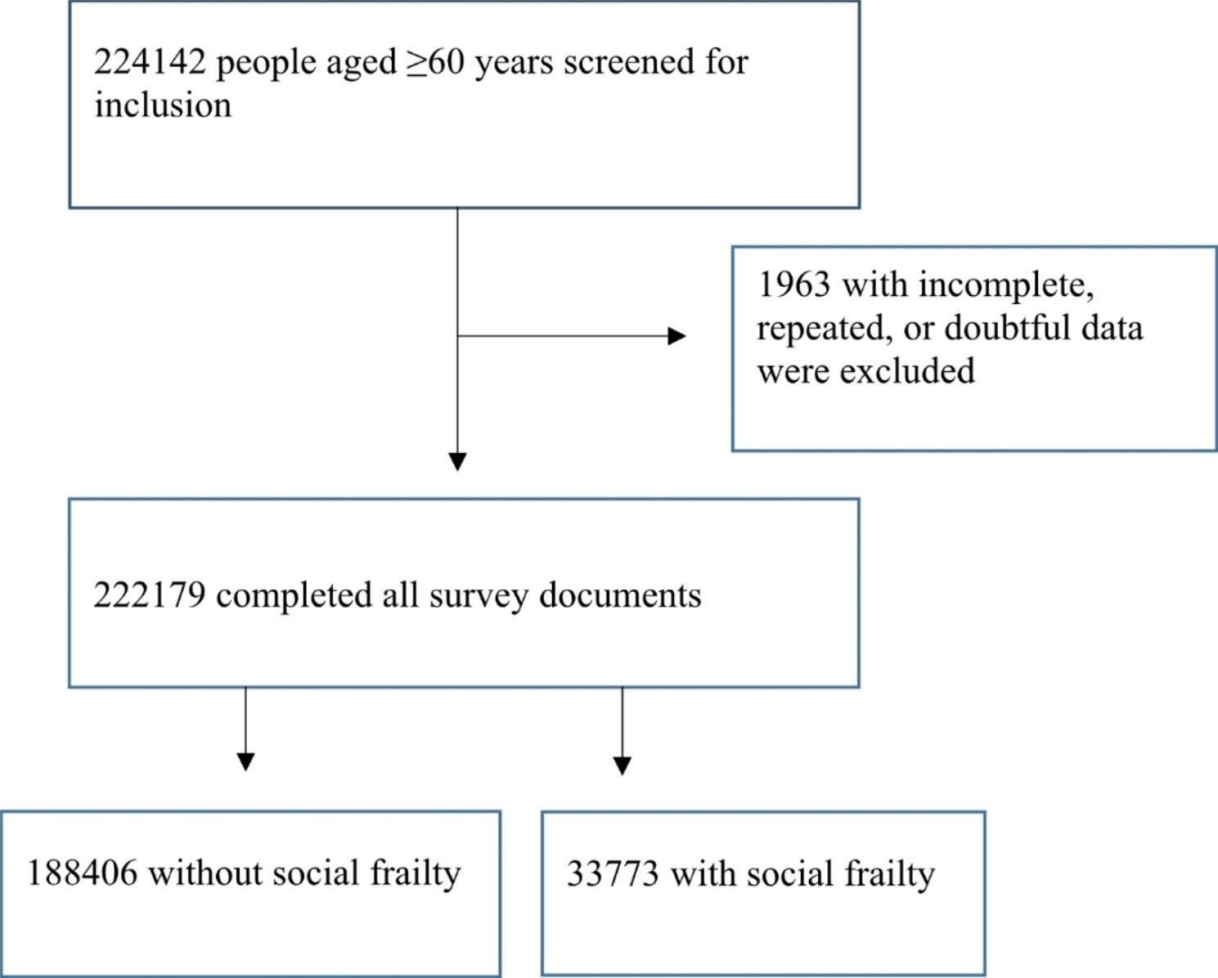
Study flowchart

**Fig. 2:**
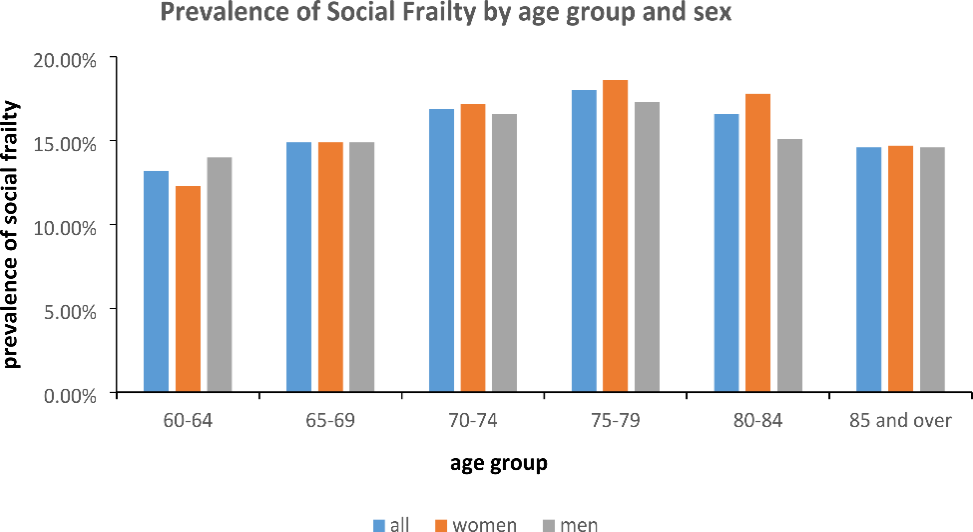
Prevalence of social frailty by age group and sex

The sample size initially planned by SSAPUR in 2015 was 223,680, and the actual number of respondents included was 224,142. After excluding 1,963 cases with missing, doubtful, or duplicate data, a total of 222,179 participants were included in this analysis. Among them, 33,773 participants (15.2%) met the criteria for social frailty, while 188,406 participants (84.8%) did not (Fig. 1). In terms of gender, no significant difference was found between the social frailty group and the non-social frailty group. However, significant differences were observed in terms of age, urban-rural distribution, ethnicity, marital status, literacy status, physical exercise participation, hospitalization within the past year, self-assessed health status, crutch or wheelchair usage, urinary and fecal incontinence, need for assistance from others, history of falls, housing satisfaction, self-assessed happiness, and the investigators’ assessment of participants’ ability to take care of themselves (Table 1). The prevalence of social frailty, based on age and gender, indicated that the highest prevalence was observed in participants aged 75–79 years (refer to Fig. 2 for details). Multivariate regression analysis revealed that age, living in an urban versus rural environment, ethnicity, marital status, number of comorbid chronic diseases, hospitalization within the past year, self-assessed health status, crutch or wheelchair usage, fecal incontinence, need for assistance from others, history of falls, housing satisfaction, self-assessed happiness, and the respondents’ ability to take care of themselves were all associated with social frailty (Table 2).

**Table 1:** Social frailty status and baseline information of the participants

		Non Social frailty n(%)	Social frailty n(%)	Total	Pearson	p
Gender	Female	98,392(84.8)	17,646(15.2)	116,038		
	Male	90,014(84.8)	16,127(15.2)	106,141	0.007	0.931
Age	60–64	63,499(86.8)	9638(13.2)	73,137		
	65–69	44,649(85.1)	7836(14.9)	52,485		
	70–74	30,741(83.1)	6240(16.9)	36,981		
	75–79	23,822(82.0)	5212(18.0)	29,034		
	80–84	15,935(83.4)	3174(16.6)	19,109		
	85 and over	9760(85.4)	1673(14.6)	11,433	518.122	0.000
Urban and rural areas	Urban	103,067(89.1)	12,593(10.9)	115,660		
	Rural	85,339(80.1)	21,180(19.9)	106,519	3481.219	0.000
Ethnicity	Han ethnic group	177,338(85.1)	31,164(14.9)	208,502		
	Non-Han ethnic group	11,068(80.9)	2609(19.1)	13,677	169.774	0.000
illiteracy status	Non-illiteracy	135,719(86.5)	21,186(13.5)	156,905		
	Illiteracy	52,687(80.7)	12,587(19.3)	65,274	1195.103	0.000
Marriage status	Spousal presence	145,704(90.4)	15,413(9.6)	161,117		
	Without spouses	42,702(69.9)	18,360(30.1)	61,062	14438.451	0.000
Physical exercise	Once a week or more	89,032(87.3)	12,989(12.7)	102,021		
	No	99,374(82.7)	20,784(17.3)	120,158	892.203	0.000
Cataract/glaucoma	Without	159,459(84.9)	28,304(15.1)	187,763		
	With	28,947(84.1)	5469(15.9)	34,416	15.044	0.000
Hypertension	Without	114,681(84.5)	21,077(15.5)	135,758		
	With	73,725(85.3)	12,696(14.7)	86,421	28.531	0.000
Heart and brain diseases	Without	135,482(85.1)	23,659(14.9)	159,141		
	With	52,924(84.0)	10,114(16.0)	63,038	48.576	0.000
Diabetes mellitus	Without	165,398(84.3)	30,918(15.7)	196,316		
	With	23,008(89.0)	2855(11.0)	25,863	393.319	0.000
Osteopathy	Without	112,470(87.7)	15,800(12.3)	128,270		
	With	75,936(80.9)	17,973(19.1)	93,909	1956.878	0.000
Cancer	Without	186,440(84.9)	33,274(15.1)	219,714		
	With	1966(79.8)	499(20.2)	2465	49.171	0.000
Lung diseases	Without	171,287(85.5)	28,939(14.5)	200,226		
	With	17,119(78.0)	4834(22.0)	21,953	878.729	0.000
Number of chronic diseases	Less than 2	103,955(86.8)	15,748(13.2)	119,703		
	2 or more	84,451(82.4)	18,025(17.6)	102,476	841.932	0.000
Hospitalization within 1 year	No	138,232(85.9)	22,624(14.1)	160,856		
	Once or more	50,174(81.8)	11,149(18.2)	61,323	583.520	0.000
Self-awareness of health	Healthy	164,459(86.5)	25,600(13.5)	190,059		
	Not healthy	23,947(74.6)	8173(25.4)	32,120	3057.065	0.000
Dentures	No	140,746(84.9)	25,121(15.1)	165,867		
	Yes	47,660(84.6)	8652(15.4)	56,312	1.566	0.211
Crutches using	No	172,606(85.1)	30,334(14.9)	202,940		
	Yes	15,800(82.1)	3439(17.9)	19,239	116.867	0.000
Wheel chairs using	No	184,880(84.8)	33,257(15.2)	218,137		
	Yes	3526(87.2)	516(12.8)	4042	18.935	0.000
Fecal incontinence	No	169,838(84.6)	30,971(15.4)	200,809		
	Yes	18,568(86.9)	2802(13.1)	21,370	80.044	0.000
Urinary incontinence	No	165,493(84.8)	29,746(15.2)	195,239		
	Yes	22,913(85.1)	4027(14.9)	26,940	1.520	0.218
Hearing aids	No	185,560(84.8)	33,277(15.2)	218,837		
	Yes	2846(85.2)	496(14.8)	3342	0.340	0.560
Diapers	No	186,667(84.8)	33,450(15.2)	220,117		
	Yes	1739(84.3)	323(15.7)	2062	0.347	0.556
Need care from others	No	161,993(85.2)	28,206(14.8)	190,199		
	Yes	26,413(82.6)	5567(17.4)	31,980	141.156	0.000
Falls	No	158,917(86.3)	25,281(13.7)	184,198		
	Yes	29,489(77.6)	8492(22.4)		1820.867	0.000
Housing satisfaction	Satisfied	166,559(86.9)	25,153(13.1)	191,712		
	Dissatisfied	21,847(71.7)	8620(28.3)	30,467	4695.088	0.000
Happiness	Happy	177,181(85.2)	30,732(14.8)	207,913		
	Unhappy	11,225(78.7)	3041(21.3)	14,266	442.328	0.000
Self-care ability	Fully independent	156,376(85.9)	25,632(14.1)	182,008		
	Dependent	32,030(79.7)	8141(20.3)	40,171	975.968	0.000

**Table 2:** Factors associated with social frailty

Variables	Groups	OR	95%CI	P value
Age	60–64	0.385	0.361-0.410	0.000
	65–69	0.393	0.369-0.419	0.000
	70–74	0.416	0.390-0.444	0.000
	75–79	0.482	0.452-0.515	0.000
	80–84	0.665	0.621-0.712	0.000
	85 and over			
Ethnicity	Non-Han ethic group	1.075	1.024–1.128	0.003
	Han ethic group			
Marriage status	Without spouses	4.874	4.743–5.009	0.000
	Spousal presence			
Urban or rural area	Rural	1.789	1.742–1.837	0.000
	Urban			
Number of chronic diseases	2 or more	1.212	1.166–1.260	0.000
	Less than 2			
Crutches using	Yes	1.059	1.011–1.109	0.016
	No			
Wheel chairs using	Yes	0.788	0.711-0.873	0.000
	No			
Diapers	Yes	1.188	1.039–1.357	0.012
	No			
Housing satisfaction	Dissatisfied	2.303	2.233–2.376	0.000
	Satisfied			
Hospitalization within 1 year	Yes	1.175	1.142–1.208	0.000
	No			
Cataract/glaucoma	Yes	1.046	1.011–1.082	0.010
	No			
Hypertension	Yes	0.869	0.842-0.896	0.000
	No			
Diabetes mellitus	Yes	0.694	0.662-0.727	0.000
	No			
Heart and brain diseases	Yes	0.955	0.924-0.986	0.005
	No			
Osteopathy	Yes	1.294	1.257–1.333	0.000
	No			
Lung diseases	Yes	1.260	1.211–1.311	0.000
	No			
Cancer	Yes	1.336	1.200-1.488	0.000
	No			
Fecal incontinence	Yes	0.736	0.703-0.772	0.000
	No			
Falls	Yes	1.350	1.308–1.392	0.000
	No			
Physical exercise	Once a week or more	0.965	0.939-0.992	0.010
	No			
Happiness	Unhappy	1.295	1.236–1.356	0.000
	Happy			
Healthy status	Unhealthy	1.696	1.633–1.761	0.000
	Healthy			
Self-care ability	Fully independent	0.869	0.837-0.901	0.000
	Dependent			
Need care from others	Yes	1.089	1.048–1.132	0.000
	No			

### Social frailty of the elderly in urban versus rural areas

**Fig. 3:**
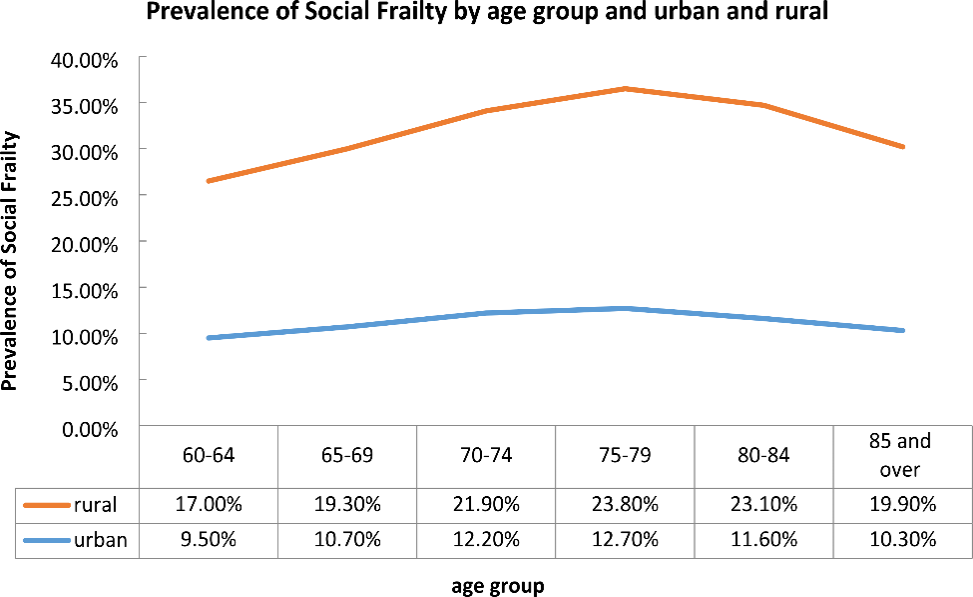
Prevalence of social frailty by age group and urban and rural area

Further analysis of the prevalence of social frailty in urban and rural populations revealed a significantly higher prevalence of social frailty among rural respondents across all age groups (Fig. 3). In urban areas, the prevalence of social frailty was higher in women compared to men (11.7% in women vs. 9.9% in men, P < 0.0001), while in rural areas, it was higher in men compared to women (19.2% in women vs. 20.6% in men, P < 0.0001). The presence of physical exercise had a significant impact on social frailty among urban participants (9.4% with physical exercise vs. 13.1% without physical exercise, P < 0.0001), but it had no effect among rural participants (19.7% with physical exercise vs. 19.9% without physical exercise, P = 0.446).

Regarding comorbid diseases, cataract/glaucoma and dentures had no effect on social frailty in urban areas (11.0% with cataract/glaucoma vs. 10.9% without cataract/glaucoma, P = 0.526; 10.9% with dentures vs. 10.8% without dentures, P = 0.364). However, a significant effect was observed in rural areas (22.3% with cataract/glaucoma vs. 19.5% without cataract/glaucoma, P < 0.0001; 21.2% with dentures vs. 19.5% without dentures, P < 0.0001). On the contrary, the use of wheelchairs had an effect on social frailty among urban older people (9.1% with wheelchair usage vs. 10.9% without, P = 0.003), but no effect was observed among rural older people (19.5% with wheelchair usage vs. 19.9% without, P = 0.750). Additional details can be found in Table 3.

**Table 3:** Analysis of social frailty by urban and rural areas

		Urban area				Rural area			
		Non-social frailty n(%)	Social frailty n(%)	Total	P	Non-social frailty n(%)	Social frailty n(%)	Total	P
Gender	Female	54,401(88.3)	7220(11.7)	61,621	0.000	43,991(80.8)	10,426(19.2)	54,417	0.000
	Male	48,666(90.1)	5373(9.9)	54,039		41,348(79.4)	10,754(20.6)	52,102	
Ethnicity	Han ethic group	98,669(89.2)	11,912(10.8)	110,581	0.000	78,669(80.3)	19,252(19.7)	97,921	0.000
	Non-Han ethic group	4398(86.6)	681(13.4)	5079		6670(77.6)	1928(22.4)	8598	
Illiteracy status	Illiteracy	21,542(84.8)	3871(15.2)	25,413	0.000	31,145(78.1)	8716(21.9)	39,861	0.000
	Non-illiteracy	81,525(90.3)	8722(9.7)	90,247		54,194(81.3)	12,464(18.7)	66,658	
Marriage status	Spousal presence	79,987(93.8)	5270(6.2)	85,257	0.000	65,717(86.6)	10,143(13.4)	75,860	0.000
	Without spouses	23,080(75.9)	7323(24.1)	30,403		19,622(64.0)	11,037(36.0)	30,659	
Age	60–64	33,775(90.5)	3544(9.5)	37,319	0.000	29,724(83.0)	6094(17.0)	35,818	0.000
	65–69	23,904(89.3)	2867(10.7)	26,771		20,745(80.7)	4969(19.3)	25,714	
	70–74	16,802(87.8)	2324(12.2)	19,126		13,939(78.1)	3916(21.9)	17,855	
	75–79	13,439(87.3)	1961(12.7)	15,400		10,383(76.2)	3251(23.8)	13,634	
	80–84	9522(88.4)	1251(11.6)	10,773		6413(76.9)	1923(23.1)	8336	
	85 and over	5625(89.7)	646(10.3)	6271		4135(80.1)	1027(19.9)	5162	
Physical exercise	Once a week or more	62,676(90.6)	6505(9.4)	69,181	0.000	26,356(80.3)	6484(19.7)	32,840	0.446
	No	40,391(86.9)	6088(13.1)	46,479		58,983(80.1)	14,696(19.9)	73,679	
Cataract/glaucoma	No	85,645(89.1)	10,432(10.9)	96,077	0.468	73,814(80.5)	17,872(19.5)	91,686	0.000
	Yes	17,422(89.0)	2161(11.0)	19,583		11,525(77.7)	3308(22.3)	14,833	
Hypertension	No	59,685(88.7)	7601(11.3)	67,286	0.000	54,996(80.3)	13,476(19.7)	68,472	0.026
	Yes	43,382(89.7)	4992(10.3)	48,374		30,343(79.8)	7704(20.2)	38,047	
Heart and brain diseases	No	72,454(89.5)	8506(10.5)	80,960	0.000	63,028(80.6)	15,153(19.4)	78,181	0.000
	Yes	30,613(88.2)	4087(11.8)	34,700		22,311(78.7)	6027(21.3)	28,338	
Diabetes mellitus	No	87,974(88.7)	11,224(11.3)	99,198	0.000	77,424(79.7)	19,694(20.3)	97,118	0.000
	Yes	15,093(91.7)	1369(8.3)	16,462		7915(84.2)	1486(15.8)	9401	
Osteopathy	No	64,855(91.2)	6293(8.8)	71,148	0.000	47,615(83.4)	9507(16.6)	57,122	0.000
	Yes	38,212(85.8)	6300(14.2)	44,512		37,724(76.4)	11,673(23.6)	49,397	
Cancer	No	101,741(89.2)	12,362(10.8)	114,103	0.000	84,699(80.2)	20,912(19.8)	105,611	0.000
	Yes	1326(85.2)	231(14.8)	1557		640(70.5)	268(29.5)	908	
Lung diseases	No	94,622(89.6)	11,018(10.4)	105,640	0.000	76,665(81.1)	17,921(18.9)	94,586	0.000
	Yes	8445(84.3)	1575(15.7)	10,020		8674(72.7)	3259(27.3)	11,933	
Dentures	No	74,912(89.1)	9201(10.9)	84,113	0.364	65,834(80.5)	15,920(19.5)	81,754	0.000
	Yes	28,155(89.2)	3392(10.8)	31,547		19,505(78.8)	5260(21.2)	24,765	
Crutching using	No	95,163(89.2)	11,502(10.8)	106,665	0.000	77,443(80.4)	18,832(19.6)	96,275	0.000
	Yes	7904(87.9)	1091(12.1)	8995		7896(77.1)	2348(22.9)	10,244	
Wheel chairs using	No	100,681(89.1)	12,354(10.9)	113,035	0.003	84,199(80.1)	20,903(19.9)	105,102	0.750
	Yes	2386(90.9)	239(9.1)	2625		1140(80.5)	277(19.5)	1417	
Hospitalization within 1 year	No	76,315(90.1)	8422(9.9)	84,737	0.000	61,917(81.3)	14,202(18.7)	76,119	0.000
	Once or more	26,752(86.5)	4171(13.5)	30,923		23,422(77.0)	6978(23.0)	30,400	
Self-awareness of healthy	Healthy	92,310(90.3)	9959(9.7)	102,269	0.000	72,149(82.2)	15,641(17.8)	87,790	0.000
	Unhealthy	10,757(80.3)	2634(19.7)	13,391		13,190(70.4)	5539(29.6)	18,729	
Fecal incontinence	No	92,414(88.9)	11,577(11.1)	103,991	0.000	77,424(80.0)	19,394(20.0)	96,818	0.000
	Yes	10,653(91.3)	1016(8.7)	11,669		7915(81.6)	1786(18.4)	9701	
Urinary incontinence	No	90,184(89.1)	11,068(10.9)	101,252	0.211	75,309(80.1)	18,678(19.9)	93,987	0.809
	Yes	12,883(89.4)	1525(10.6)	14,408		10,030(80.0)	2502(20.0)	12,532	
Supporting supplies									
Hearing aids	No	101,353(89.1)	12,384(10.9)	113,737	0.978	84,207(80.1)	20,893(19.9)	105,100	0.745
	Yes	1714(89.1)	209(10.9)	1923		1132(79.8)	287(20.2)	1419	
Diapers	No	101,911(89.1)	12,444(10.9)	114,355	0.537	84,756(80.1)	21,006(19.9)	105,762	0.032
	Yes	1156(88.6)	149(11.4)	1305		583(77.0)	174(23.0)	757	
Need care from others	No	89,481(89.3)	10,700(10.7)	100,181	0.000	72,512(80.6)	17,506(19.4)	90,018	0.000
	Yes	13,586(87.8)	1893(12.2)	15,479		12,827(77.7)	3674(22.3)	16,501	
Number of chronic diseases	Less than 2	56,715(90.5)	5964(9.5)	62,679	0.000	47,240(82.8)	9784(17.2)	57,024	0.000
	2 or more	46,352(87.5)	6629(12.5)	52,981		38,099(77.0)	11,396(23.0)	49,495	
Falls	No	89,258(90.1)	9769(9.9)	99,027	0.000	69,659(81.8)	15,512(18.2)	85,171	0.000
	Yes	13,809(83.0)	2824(17.0)	16,633		15,680(73.4)	5668(26.6)	21,348	
Housing satisfaction	Satisfied	92,209(90.5)	9727(9.5)	101,936	0.000	74,350(82.8)	15,426(17.2)	89,776	0.000
	Dissatisfied	10,858(79.1)	2866(20.9)	13,724		10,989(65.6)	5754(34.4)	16,743	
Happiness	Happy	98,586(89.4)	11,743(10.6)	110,329	0.000	78,595(80.5)	18,989(19.5)	97,584	0.000
	Unhappy	4481(84.1)	850(15.9)	5331		6744(75.5)	2191(24.5)	8935	
Self-care ability	Fully independent	87,737(89.8)	9914(10.2)	97,651	0.000	68,639(81.4)	15,718(18.6)	84,357	0.000
	Dependent	15,330(85.1)	2679(14.9)	18,009		16,700(75.4)	5462(24.6)	22,162	

Regression analyses were conducted separately for urban and rural populations. The multivariate regression analysis showed that age, ethnicity, marital status, number of comorbid chronic diseases, hospitalization within the past year, self-assessed health status, wheelchair usage, fecal incontinence, need for assistance from others, history of falls, housing satisfaction, self-assessed happiness, and respondents’ ability to take care of themselves were all associated with social frailty in both urban and rural areas. However, the effects of illiteracy, cataract/glaucoma, dentures, crutch usage, and physical exercise on social frailty were inconsistent between urban and rural areas (see details in Table 4).

**Table 4:** Factors associated with social frailty by urban area and rural area

Variables	Groups	urban area			rural area		
		OR	95%CI	P	OR	95%CI	P
Gender	Female	1.423	1.362–1.487	0.000	**1.451**	1.401–1.503	0.000
	Male						
Ethnicity	Non-Han ethic group	1.124	1.028–1.229	0.011	1.076	1.016–1.140	0.012
	Han ethic group						
Age	60–64	0.299	0.269-0.329	0.000	0.443	0.408-0.482	0.000
	65–69	0.308	0.279-0.341	0.000	0.449	0.413-0.488	0.000
	70–74	0.338	0.306-0.374	0.000	0.467	0.430-0.509	0.000
	75–79	0.429	0.388-0.475	0.000	0.518	0.476-0.564	0.000
	80–84	0.625	0.562-0.694	0.000	0.688	0.629-0.753	0.000
	85 and over						
Marriage status	Without spouses	6.177	5.909–6.458	0.000	4.573	4.410–4.742	0.000
	Spousal presence						
illiteracy status	Illiterale	1.240	1.181–1.302	0.000	1.022	0.985-1.061	0.250
	Non-illiterale						
Physical exercise	No	1.103	1.057–1.150	0.000	0.863	0.833-0.894	0.000
	Once a week or more						
Cataract/glaucoma	Yes	0.996	0.945 − 1.050	0.887	1.095	1.046–1.146	0.000
	No						
Number of chronic diseases	2 or more	1.219	1.145–1.298	0.000	1.206	1.148–1.266	0.000
	Less than 2						
Crutches using	Yes	1.027	0.949-1.111	0.506	1.076	1.016–1.016	0.013
	No						
hearing-aids	Yes	0.994	0.851-1.161	0.941	0.958	0.833-1.012	0.550
	No						
denture	Yes	0.962	0.919-1.006	0.087	1.084	1.043–1.125	0.000
	No						
Wheel chairs using	Yes	0.720	0.617-0.841	0.000	0.847	0.730-0.982	0.028
	No						
Diapers	Yes	1.131	0.932-1.372	0.214	1.201	0.994-1.451	0.058
	No						
Housing satisfaction	Dissatisfied	2.261	2.151–2.378	0.000	2.301	2.212–2.394	0.000
	Satisfied						
Hospitalization within 1 year	Yes	1.216	1.163–1.272	0.000	1.140	1.099–1.182	0.000
	No						
Hypertension	Yes	0.815	0.776-0.855	0.000	0.935	0.898-0.974	0.001
	No						
Diabetes mellitus	Yes	0.703	0.657-0.752	0.000	0.728	0.681-0.778	0.000
	No						
Heart and brain diseases	Yes	0.964	0.916-1.015	0.160	0.988	0.947 − 1.030	0.568
	No						
Osteopathy	Yes	1.345	1.283–1.410	0.000	1.334	1.284–1.385	0.000
	No						
Lung diseases	Yes	1.199	1.122–1.280	0.000	1.239	1.178–1.303	0.000
	No						
Cancer	Yes	1.180	1.012–1.377	0.035	1.563	1.338–1.825	0.000
	No						
Fecal incontinence	Yes	0.650	0.589-0.717	0.000	0.821	0.763-0.884	0.000
	No						
Urinary incontinence	Yes	1.065	0.978 − 1.160	0.148	0.897	0.841-0.956	0.001
	No						
Falls	Yes	1.434	1.362–1.509	0.000	1.328	1.277–1.382	0.000
	No						
Happiness	Unhappy	1.429	1.316–1.553	0.000	1.225	1.159–1.294	0.000
	Happy						
Self-awareness of healthy	Unhealthy	1.803	1.692–1.921	0.000	1.631	1.558–1.710	0.000
	Healthy						
Self-care ability	Fully independent	0.858	0.806-0.913	0.000	0.872	0.832-0.913	0.000
	Dependent						
Need care from others	yes	1.098	1.030–1.171	0.004	1.074	1.023–1.127	0.004
	No						

## Discussion

To the best of our knowledge, this study represents the largest survey conducted to date on social frailty among older adults in urban and rural areas of China. The findings of this study provide valuable insights into the prevalence of social frailty among older adults in China and shed light on the health risk factors and socioeconomic factors associated with its occurrence.

### Social frailty versus non-social frailty

This cross-sectional study encompasses a larger sample size compared to previous studies conducted in China. Earlier small-scale studies reported a social frailty prevalence of 7.7% in the Chinese population [6]. However, our study revealed a higher prevalence of 15.2%, which is significantly lower than that reported in Korea (44.7%) but falls between the rates observed in Singapore (18.4%) and Japan (11.1%) [7–10]. It is important to note that our study included community-dwelling older adults from both rural and urban areas, distinguishing it from previous research. Consequently, the differences between this study and earlier ones primarily lie in the composition of the social frailty questionnaire and the study population.

Our findings demonstrate that the prevalence of social frailty varies across age groups. It gradually increases with age up to 80 years, after which it starts to decline. Notably, the highest prevalence of social frailty was observed among participants aged 75–79 years, deviating from previous studies. In 2018, the average life expectancy in China was 77 years per capita.It remains uncertain whether the coincidence of the highest prevalence of social frailty in the 75–79 age group aligns with average life expectancy. Social frailty has been associated with reduced dietary intake, poor diet quality, and inadequate nutrition among community-dwelling older men [7–10, 20, 21, 37, 43, 44]. Furthermore, it serves as a predictor for physical frailty, cognitive decline, hospitalization, and mortality in this population. The decrease in social frailty prevalence among those aged 80 and above may be due to the higher survival rate of older adults without social frailty and their increased likelihood of having companions, reduced solitary living, and more social interactions, thus mitigating the prevalence of social frailty.

It is worth noting that the age-specific prevalence of social frailty differs from that of physical frailty, which consistently increases with age. Physical frailty is more prevalent among women than men in advanced age, while the highest prevalence of social frailty in this study was observed in the 75–79 age group and did not significantly differ by gender. Additionally, low educational levels have been associated with a higher incidence of physical frailty in previous studies [9, 11, 33, 35, 37], but in our multivariate analysis, educational level did not show a significant relationship with social frailty.Analyze the reason, perhaps it is because that other factors such as urban-rural disparities, housing satisfaction and marriage status had a more significant impact on social frailty in our study. The association between educational level and social frailty in China need further research.

In this study, we discovered a significant association between social frailty and the presence of a spouse. Participants with spouses exhibited a significantly lower incidence of social frailty, while not having a spouse was identified as a risk factor for social frailty [45–49]. Older individuals without spouses often find themselves performing tasks independently and experiencing limited communication and social connections. These limitations can have adverse effects on their physical, cognitive, and social well-being. Living alone is common among older individuals without spouses, leading to reduced social participation and an increased risk of functional decline. Previous studies have consistently shown that being married is significantly associated with a decreased risk of frailty in older adults [50, 51]. This can be attributed to the increased social support and reduced engagement in risky behaviors among married individuals. Moreover, older adults with spouses tend to have better physical health. Marriage serves as a crucial source of social support, especially when social engagement becomes limited in later life. The institution of marriage provides various benefits, including access to marital resources and assets, monitoring of each other’s health and behaviors, and the formation of social bonds. Married individuals have greater access to social, psychological, and economic resources compared to singles, all of which contribute to better health and longevity.

Furthermore, we found a significant association between housing satisfaction and social frailty. While previous studies on the correlation between housing/relocation and social frailty are limited [52], our findings shed light on the subject. China, being a vast country with disparities in social development and conditions between urban and rural areas, exhibits diverse housing arrangements such as self-built rural houses, rented apartments, living with children, and, to a lesser extent, residing in nursing homes. Chinese older adults primarily reside in their own homes, and factors like private housing, presence of an elevator, and availability of suitable sanitary facilities can pose inconveniences and safety concerns for the elderly. We observed a noteworthy correlation between housing dissatisfaction and social frailty. Housing satisfaction, favorable living environments, and suitable housing facilities that promote active living can enhance the social activities and interactions of older adults. Additionally, elderly respondents who reported housing satisfaction generally had better financial circumstances, which is another factor associated with reduced social frailty. While the government has initiated efforts to improve housing conditions for the elderly, further research is needed to fully understand the relationship between housing status and social frailty.

### Social frailty of the older people in urban versus rural areas

Our survey reveals a significant disparity in social frailty between urban and rural areas in mainland China, with a considerably higher prevalence observed in rural populations compared to urban populations. Educational opportunities, economic status, happiness levels, depression scores, and exercise scores are unevenly distributed between rural and urban regions in China, with rural older adults lagging behind their urban counterparts in these aspects [53]. Consequently, older individuals in rural areas lack the social resources necessary to meet their basic social needs, engage in social behaviors and activities, and possess self-management skills, resulting in a higher incidence of social frailty compared to urban areas. This discrepancy can be attributed to the significant economic development gap between urban and rural areas in China, as well as the inadequate infrastructure in rural regions [53, 54]. Moreover, older adults in rural areas often engage in physically demanding work such as farming, which can lead to health issues such as overexertion and joint diseases. As a result, physical activity has not been as effective in reducing social frailty among rural older adults as it has been for their urban counterparts. [53].

Another contributing factor is the diminished spiritual support from adult children due to the migration of the rural labor force. Many older individuals in rural areas have children who are far away from home and have less time available to provide spiritual support [53]. This, to some extent, affects the health of older adults in rural areas. Additionally, the distribution of medical resources in China is currently imbalanced, with tertiary hospitals primarily concentrated in urban areas. Rural regions suffer from a shortage of medical resources, including doctors and nurses, and the quality of healthcare services in rural primary care institutions needs improvement [54]. These factors may contribute to poorer health outcomes among older adults in rural areas.

Furthermore, the prevalence of social frailty is higher among rural women than men, whereas no gender difference exists in urban areas. This discrepancy may be rooted in historical preferences for sons in Chinese society and the challenges faced by older women, who not only had to cope with employment pressures during their younger years but also took on significant domestic responsibilities and cared for their partners and third-generation grandchildren as they entered old age.

The disease affects the physical health of the older people, leading to poverty and reduced social interaction. Previous studies have shown that urban older individuals generally have better health status compared to rural older individuals [53]. In this study, cataract/glaucoma is associated with social frailty among rural older individuals but not among urban older individuals. If properly treated, cataract/glaucoma do not influence the visual acuity, but if not treated in a timely manner, cataract/glaucoma can lead to blind and visually impaired. Impaired vision affects social interaction and participation in social activities. So rural older people with cataract/glaucoma had a higher risk of social frailty. In China rural areas, older people may do not seek medical attention in a timely manner. This difference may be attributed to the fact that older people in rural areas primarily engage in physical work, the uneven distribution of medical resources, and their tendency to delay seeking medical treatment for various reasons, resulting in the worsening of their conditions. Our research further confirms the findings of previous studies that chronic diseases is associated with social frailty [4, 5].

One of the strengths of this study is its large sample size. However, it is important to note that large sample sizes can sometimes yield statistical differences that may not necessarily indicate significant associations between groups. Therefore, special attention is required when interpreting the statistical results presented in this paper. Prospective studies are necessary to establish causal relationships between socioeconomic factors and frailty, and further research is needed to uncover the specific mechanisms underlying the association between socioeconomic factors and frailty among older people.

Nevertheless, this study also has some limitations. Firstly, all the data collected were self-reported and may be susceptible to memory bias. Secondly, as cross-sectional data were used, it was not possible to explore causality. This aspect should be addressed in future prospective studies. Thirdly, while this study identified several factors associated with social frailty, only some of them have been discussed. Further analysis of other relevant factors is necessary in future research. Additionally, there is a need for further studies to develop effective intervention strategies for social frailty aimed at improving and enhancing healthy aging.

## Conclusions

We employed the HALFE social frailty index to investigate social frailty among elderly individuals in both urban and rural areas of China. Our study revealed an overall incidence of 15.2%, with the highest occurrence observed among individuals aged 75–79. Numerous factors, both modifiable and non-modifiable, are associated with social frailty. Specifically, the presence of a spouse, housing satisfaction, health status, and differences in urban-rural residential settings were found to have significant associations with social frailty. Moreover, we found that the prevalence of social frailty is notably higher in rural areas compared to urban areas.

### Electronic supplementary material

Below is the link to the electronic supplementary material.

Supplementary Material 1

Supplementary Material 2

## Data Availability

Data will be available upon request from the corresponding author.
