# Supplementary material for: Prevalence of social frailty and its associated factors in the older Chinese population: a national cross-sectional study
Source: BMC Geriatr. 2023 Sep 1;23:532. doi: 10.1186/s12877-023-04241-1 (PMC10474699; doi:10.1186/s12877-023-04241-1)
Supplement: Supplementary file 2 — Supplementary Material 2 [file 12877_2023_4241_MOESM2_ESM.docx]

Table S1. Social frailty status and baseline information of the participants

|  |  | Non-social frailty | | Social frailty | | Overall | | Pearson | Fisher's exact test |
| --- | --- | --- | --- | --- | --- | --- | --- | --- | --- |
|  |  | n | % | n | % | n | % |  |  |
| Gender | Female | 98392 | 84.8 | 17646 | 15.2 | 116038 |  |  |  |
|  | Male | 90014 | 84.8 | 16127 | 15.2 | 106141 |  | .007 | .931 |
| Age |  |  |  |  |  |  |  |  |  |
|  | 60-64 | 63499 | 86.8 | 9638 | 13.2 | 73137 |  |  |  |
|  | 65-69 | 44649 | 85.1 | 7836 | 14.9 | 52485 |  |  |  |
|  | 70-74 | 30741 | 83.1 | 6240 | 16.9 | 36981 |  |  |  |
|  | 75-79 | 23822 | 82.0 | 5212 | 18.0 | 29034 |  |  |  |
|  | 80-84 | 15935 | 83.4 | 3174 | 16.6 | 19109 |  |  |  |
|  | 85 and over | 9760 | 85.4 | 1673 | 14.6 | 11433 |  | 518.122 | .000 |
| Urban and rural areas | Urban | 103067 | 89.1 | 12593 | 10.9 | 115660 |  |  |  |
|  | Rural | 85339 | 80.1 | 21180 | 19.9 | 106519 |  | 3481.219 | .000 |
| Ethnicity | Han ethnic group | 177338 | 85.1 | 31164 | 14.9 | 208502 | 100 |  |  |
|  | Non-Han ethnic group | 11068 | 80.9 | 2609 | 19.1 | 13677 | 100 | 169.774 | .000 |
| Educational level | Non-illiteracy | 135719 | 86.5 | 21186 | 13.5 | 156905 |  |  |  |
|  | Illiteracy | 52687 | 80.7 | 12587 | 19.3 | 65274 |  | 1195.103 | .000 |
| Marriage status | Spousal presence | 145704 | 90.4 | 15413 | 9.6 | 161117 |  |  |  |
|  | Single | 42702 | 69.9 | 18360 | 30.1 | 61062 |  | 14438.451 | .000 |
| Physical exercise | Once a week or more | 89032 | 87.3 | 12989 | 12.7 | 102021 |  |  |  |
|  | No | 99374 | 82.7 | 20784 | 17.3 | 120158 |  | 892.203 | .000 |
|  |  |  |  |  |  |  |  |  |  |
| Cataract/ glaucoma | Without | 159459 | 84.9 | 28304 | 15.1 | 187763 |  |  |  |
|  | With | 28947 | 84.1 | 5469 | 15.9 | 34416 |  | 15.044 | .000 |
| Hypertension | Without | 114681 | 84.5 | 21077 | 15.5 | 135758 |  |  |  |
|  | With | 73725 | 85.3 | 12696 | 14.7 | 86421 |  | 28.531 | .000 |
| Heart and brain diseases | Without | 135482 | 85.1 | 23659 | 14.9 | 159141 |  |  |  |
|  | With | 52924 | 84.0 | 10114 | 16.0 | 63038 |  | 48.576 | .000 |
| Diabetes mellitus | Without | 165398 | 84.3 | 30918 | 15.7 | 196316 |  |  |  |
|  | With | 23008 | 89.0 | 2855 | 11.0 | 25863 |  | 393.319 | .000 |
| Osteopathy | Without | 112470 | 87.7 | 15800 | 12.3 | 128270 |  |  |  |
|  | With | 75936 | 80.9 | 17973 | 19.1 | 93909 |  | 1956.878 | .000 |
| Cancer | Without | 186440 | 84.9 | 33274 | 15.1 | 219714 |  |  |  |
|  | With | 1966 | 79.8 | 499 | 20.2 | 2465 |  | 49.171 | .000 |
| Lung diseases | Without | 171287 | 85.5 | 28939 | 14.5 | 200226 |  |  |  |
|  | With | 17119 | 78.0 | 4834 | 22.0 | 21953 |  | 878.729 | .000 |
| Number of chronic diseases | Less than 2 | 103955 | 86.8 | 15748 | 13.2 | 119703 |  |  |  |
|  | 2 or more | 84451 | 82.4 | 18025 | 17.6 | 102476 |  | 841.932 | .000 |
| Hospitalization within 1 year | No | 138232 | 85.9 | 22624 | 14.1 | 160856 |  |  |  |
|  | Once or more | 50174 | 81.8 | 11149 | 18.2 | 61323 |  | 583.520 | .000 |
| Self-awareness of health | Healthy | 164459 | 86.5 | 25600 | 13.5 | 190059 |  |  |  |
|  | Not healthy | 23947 | 74.6 | 8173 | 25.4 | 32120 |  | 3057.065 | .000 |
|  |  |  |  |  |  |  |  |  |  |
|  | | | | | | | | | |
| Dentures | No | 140746 | 84.9 | 25121 | 15.1 | 165867 |  |  |  |
|  | Yes | 47660 | 84.6 | 8652 | 15.4 | 56312 |  | 1.566 | .211 |
| Crutches using | No | 172606 | 85.1 | 30334 | 14.9 | 202940 |  |  |  |
|  | Yes | 15800 | 82.1 | 3439 | 17.9 | 19239 |  | 116.867 | .000 |
| Wheel chairs using | No | 184880 | 84.8 | 33257 | 15.2 | 218137 |  |  |  |
|  | Yes | 3526 | 87.2 | 516 | 12.8 | 4042 |  | 18.935 | .000 |
| Fecal incontinence | No | 169838 | 84.6 | 30971 | 15.4 | 200809 |  |  |  |
|  | Yes | 18568 | 86.9 | 2802 | 13.1 | 21370 |  | 80.044 | .000 |
| Urinary incontinence | No | 165493 | 84.8 | 29746 | 15.2 | 195239 |  |  |  |
|  | Yes | 22913 | 85.1 | 4027 | 14.9 | 26940 |  | 1.520 | .218 |
| Hearing aids | No | 185560 | 84.8 | 33277 | 15.2 | 218837 |  |  |  |
|  | Yes | 2846 | 85.2 | 496 | 14.8 | 3342 |  | .340 | .560 |
| Diapers | No | 186667 | 84.8 | 33450 | 15.2 | 220117 |  |  |  |
|  | Yes | 1739 | 84.3 | 323 | 15.7 | 2062 |  | .347 | .556 |
| Need care from others | No | 161993 | 85.2 | 28206 | 14.8 | 190199 |  |  |  |
|  | Yes | 26413 | 82.6 | 5567 | 17.4 | 31980 |  | 141.156 | .000 |
|  |  |  |  |  |  |  |  |  |  |
| Falls | No | 158917 | 86.3 | 25281 | 13.7 | 184198 |  |  |  |
|  | Yes | 29489 | 77.6 | 8492 | 22.4 |  |  | 1820.867 | .000 |
| Housing satisfaction | Satisfied | 166559 | 86.9 | 25153 | 13.1 | 191712 |  |  |  |
|  | Dissatisfied | 21847 | 71.7 | 8620 | 28.3 | 30467 |  | 4695.088 | .000 |
|  |  |  |  |  |  |  |  |  |  |
| Happiness | Happy | 177181 | 85.2 | 30732 | 14.8 | 207913 |  |  |  |
|  | Unhappy | 11225 | 78.7 | 3041 | 21.3 | 14266 |  | 442.328 | .000 |
|  |  |  |  |  |  |  |  |  |  |
| Self-care ability | Fully independent | 156376 | 85.9 | 25632 | 14.1 | 182008 |  |  |  |
|  | Dependent | 32030 | 79.7 | 8141 | 20.3 | 40171 |  | 975.968 | .000 |
